# Family functioning, parenting stress and quality of life in mothers and fathers of Polish children with high functioning autism or Asperger syndrome

**DOI:** 10.1371/journal.pone.0186536

**Published:** 2017-10-16

**Authors:** Ewa Pisula, Anna Porębowicz-Dörsmann

**Affiliations:** Faculty of Psychology, University of Warsaw, Warsaw, Poland; Universite de Bretagne Occidentale, FRANCE

## Abstract

The aim of the study was to investigate the perception of the family functioning in parents of children with autism spectrum disorders (ASD) with normal-range intelligence and the relationships between family functioning, parenting stress and quality of life. Dyads of parents of children with ASD without intellectual disability and parents of typically developing children (controls) completed a set of self-report questionnaires. Parents of children with ASD reported lower functioning of the family as a whole and their own functioning as family members; they exhibited higher levels of parenting stress and lower quality of life. Mothers of children with ASD experienced more stress in personal domain than fathers. Relationships between family functioning, parenting stress and quality of life have been established. There were also moderate to strong correlations in mother-father dyads between their assessments of family functioning, parenting stress and QoL in social relationships and environmental domains.

## Introduction

Parents of children with autism spectrum disorders (ASD) face multiple challenges due to their child's developmental difficulties [1–4]. However, although individuals with ASD are a highly heterogeneous group, they tend to be treated “as one homogeneous category” ([5] p. 638) in much of the research on their parents. The need to conduct studies on less heterogeneous subgroups of families and to control for a variety of factors that shape parental adjustment, including child and family characteristics, has been already emphasized [6]. Considering that in recent years the number of children with ASD with normal-range intelligence has been growing [7–9], it seems necessary to seek a better understanding of the factors related to the adjustment of parents of this group of children.

Parents of children with ASD without intellectual disability may experience many problems related to child care [10–12], and can even exhibit higher levels of stress than the parents of children with ASD and intellectual disability [13–14]. Difficulties in the adjustment of parents of children with ASD with normal-range intelligence may be compounded by the fact that these children rarely receive a diagnosis before the age of 4 [15], and the scope and nature of their developmental problems often eludes precise characterizations. Parenting stress may result from the child’s social and behavioral problems, alienation and experience of bullying, as well as deficits in planning and organizing his/her own activities and being less independent than peers [12, 16–18]. A number of these children suffer from emotional problems, depression and anxiety, which may also contribute to their parents’ stress [18–20]. The non-obvious character of the disorder leads to a lack of understanding for both the children's and parent's problems, and to questioning of parental competences [18], and the challenges stemming from child rearing can significantly affect family life and expose it to crises [21].

According to the ABCX model [22], one of the key variables for parental adjustment is family functioning, counted among the resources facilitating adjustment (i.e. [23–26]). It has been demonstrated that family cohesion and quality of marriage are related to parenting stress and depressive symptoms in parents [27,28]. Family commitment, challenge, cohesion, expression and marital support are recognized as the predictors of family quality of life [29]. Research findings have corroborated the relationship between the functioning level of a child with ASD (including autism symptom severity, IQ, communication skills) and family system characteristics and parental satisfaction with family functioning [30,31]. However, there have been very few studies on the families with children with ASD without intellectual disability and relationships between their functioning and parental adjustment. It has been found that parents of these children experience marital difficulties, report lower satisfaction with their marriage and exhibit poorer dyadic adjustment in comparison to parents with children without any disability [32]. In comparison to families of children with learning disabilities and control groups, they perceived their family relations as less conducive to the open expression of emotions, with lower levels of mutual support, and reported their families as more organized and with higher system maintenance orientation [33].

Most studies on the relationship between family functioning and the level of adjustment of parents raising children with ASD are conducted on mothers. Considering differences between mothers’ and fathers’ levels of involvement in care tasks [34], taking parents’ gender into account when exploring variables affecting adjustment seems necessary. Mothers of children with ASD are less satisfied with marriage and less positive about their family’s adjustment to taking care of the child than mothers of children with Down syndrome and typically developing children, while no such differences in the case of fathers of children with autism were found [35,36]. The results of studies on parental adjustment in relation to parent's gender are inconsistent. Some of them have shown that parenting stress is higher in mothers than in fathers [17,29,37,38], while other researchers have detected no such differences [1,2,39] or even found that fathers experienced more stress [14]. Mothers report poorer mental and physical health, as well as other aspects of QoL [29,40–42]. In a comprehensive analysis of predictors of stress and QoL in parents of children with ASD, McStay and colleagues [29] found no gender differences. Similar reasults were found by Dardas and Ahmad [43] from a sample from Arab country. It should be stressed however, that only a few studies have been focused on the situation of mothers and fathers of children with ASD with normal-range intelligence [17,40], and links between QoL and the functioning of the family with a child with ASD [42].

### The current study

This study aims to investigate how Polish mothers and fathers of children with ASD without intellectual disability perceive the functioning of their families and to explore relationships between family functioning, parental stress and quality of life. There is no governmental program of early detection, diagnosing and supporting children with ASD, currently available in Poland. Although there is some progress in supporting children with ASD and they receive governmental educational subsidies, families of children with ASD with normal-range intelligence have still limited access to professional support. The diagnostic, educational and therapeutic services are offered mostly by centers run by non-governmental organizations or by private institutions. Due to economical discrepancies, this type of treatment is available only to a minority of children with ASD [44,45]. Parents are extensively involved in arranging support for their child. Because of the limited knowledge about autism in society at large, they often experience a lack of understanding from others and stigmatization. This seems to be common characteristic of the situation of parents of children with ASD in many countries. The burden of coordinating, advocating for, and making decisions about treatment often falls on parents [46], which may result in limiting their professional activity [47,48] and loss of family income [49]. Parents experience also stigmatization in relation to their child's unusual development and behavior [50]. Therefore, the information on parental adjustment collected in Poland may enhance general knowledge about the situation of parents of children with ASD without intellectual disability.

In the present study family functioning will be treated as one of the resources important for parental adjustment. It will be construed according to the Process Model of Family Functioning [51]. The model takes into account both the individual functioning of each family member and evaluation of the family as a whole. According to the authors, the key measure of family health is its ability to successfully adjust to stress. The model has been used in studies of families of children and adolescents with various problems [52,53]. By including multiple dimensions of family functioning and placing emphasis on coping in a crisis, the model can also be useful in studies on the adjustment of parents of children with ASD.

The measures of the adjustment will include parenting stress and quality of life. Although these phenomena are undoubtedly interrelated, they can be analysed as distinct variables (see for example [54]) in order to better understand the relation of each one to family functioning.

The study will involve dyads of parents raising a child together, which creates the unique opportunity to calculate the correlations between mothers’ and fathers’ assessments of family functioning, as well as the level of parenting stress and quality of life. The results will be compared with a group of parents of typically developing children matched for family system characteristics.

The following research questions and hypotheses have been put forward:

1. Do mothers and fathers of children with ASD without intellectual disability differ from mothers and fathers of typically developing children in their assessments of family functioning, level of parenting stress and subjective quality of life?
  We expected that the assessments of family functioning and reported quality of life would be lower in parents of children with ASD (H1), and the level of parenting stress higher compared to parents of typically developing children (H2). We also expected that between-groups differences would differ depending on parents’ gender (H3).
2. Does family functioning predict parenting stress and quality of life?
  We expected that family functioning would predict the level of stress in parents (negative correlations) (H4) and parental quality of life (positive relationship) (H5).
3. In terms of family functioning, parenting stress and quality of life–are there correlations between the results of mothers and fathers within parental dyads?
  As there are no clear theoretical grounds and only a handful of previous reports on the correlations of parental assessments of family relations, no hypotheses were advanced for this research question.

## Method

### Participants

The study included two groups of parents (N = 202): parents of ADS children (49 mother-father dyads) and parents of typically developing children (52 mother-father dyads). In the parents of children with ASD group the inclusion criteria for the primary caregivers were as follows: (a) their child had a medical diagnosis of Asperger syndrome or childhood autism according to ICD-10 [55] criteria; (b) their child had no intellectual disability; c) their child lived at home with them; (d) their child was between 5 and 17 years old; (e) their child attended a mainstream or inclusive school; (f) no concomitant conditions in children with ASD; (g) no developmental disorders or serious health problems in other children in the family; (h) the parents were partners and living together; (i) both of them completed the questionnaires; (j) both of them were biological parents of the child. From now on this group will be described as ASD group.

Parents of typically developing children were included in the current study if: (a) none of their children had received a diagnosis of any developmental disorders, intellectual disability or serious medical problems; (b) at least one of their children was living at home with them; (c) this child was between 5 and 17 years old; (d) the parents were partners and living together; (e) both of them completed the questionnaires; (f) both of them were biological parents of the child. Demographic characteristics of participants are presented in Table 1.

**Table 1:** Demographic characteristics.

Demographic characteristics	ASD		Controls	
	*%*	M (SD)	*%*	M (SD)
Family				
Place of residence				
Country	38.8		36.5	
Town to 100 thous. of citizens	25.5		19.2	
City from 100 to 500 thous.	6.1		7.8	
City more than 500 thous.	29.6		36.5	
Income per person				
Less than 500 PLN	14.3		11.5	
500–1000 PLN	24.5		25	
1000–1500 PLN	22.4		19.3	
More than 1500 PLN	38.8		44.2	
Number of children		1.84 (0.85)		2.15 (0.83)
1	38.8		20.2	
2	42.9		49	
3	16.3		25	
4 or 5	2.0		5.8	
Mother				
Age (in years)		39.56 (5.50)		39.94 (5.82)
Education level				
Basic or vocational	16.7		20.4	
Secondary / higher	83.3		79.6	
Father				
Age (in years)		41.87 (5.17)		41.62 (7.08)
Education level				
Basic or vocational	7.7		25	
Secondary / higher	92.3		75	
Child				
Gender				
Male	87.8		88.5	
Female	12.2		11.5	
Age (in years)		10.24 (3.24)		10.21 (3.75)
Diagnosis				
Asperger disorder	79.6			
Childhood autism	20.4			

ASD–autism spectrum disorders

No significant differences between groups of parents were found in most demographic variables (parents’ age, level of education, place of residence, family income, child's age and gender). There were differences in the number of children in the family (chi^2^ = 15.99, df = 4, p < .01): there were more families with children with ASD that had only one child, than in the control group.

### Measures and procedures

#### Instruments

The questionnaire used to measure family functioning was FAM III (*Family Assessment Measure III*) [51], Polish adaptation by Beauvale, de Barbaro, Namysłowska and Furgał [56]. It is a self-report instrument, based on the process model of family functioning [51]. It consists of three scales: a General Scale (40 items), in which family members evaluate the family as a system, a Dyadic Relationships Scale (28 items), in which they assess their relationship with their partner, and a Self-Rating Scale (28 items), measuring individuals’ perceptions of their functioning in the family.

Participants respond to each statement on a four-point Likert scale (0 –*strongly agree*, *1 –agree*, *2 –disagree*, *3 –strongly disagree*). Scores in each subscale are calculated as an arithmetic mean. Higher scores indicate greater family dysfunction. Mean scores in each subscale fall between 0 and 3. Lower scores in individual subscales indicate better family functioning. The questionnaire reliability expressed as Cronbach alpha coefficients ranged from .68 (for Self-Rating Scale) to .80 (for Dyadic Relationships scale).

Parenting stress was measured using the short form of the Questionnaire of Resources and Stress for Families with Chronically Ill or Handicapped Members (QRS–S) [57]. The Polish version adapted by Pisula [58] was used in the present study. The instrument measures three main areas of stress: child problems (18 items), personal problems (30 items) and family problems (18 items). For each of the 66 items participants circle one of two responses: True/False. Higher scores indicate higher stress. In the present study the total score (sum of points for the whole questionnaire) and scores in the three aforementioned scales were analysed. The reliability of the scale is satisfactory. Cronbach's alpha coefficients ranged from .70 (for Child problems scale) to .89 (Total score).

The Quality of Life Questionnaire was used (WHOQoL-BREF) [59]. It consists of 24 questions to which participants respond on a five-point Likert scale. Scores reflect subjective assessments of QoL in four domains: Physical health (7 items), Psychological (6 items), Social relationships (3 items) and Environmental (8 items). Higher scores indicate better assessment of QoL. Scores for individual domains are calculated as the arithmetic mean of the items belonging to a given domain, and then the result is multiplied by four (to obtain scores comparable with the WHOQoL full version). Polish version of the questionnaire was used [60]. Cronbach's alpha coefficients for individual scales in the present study ranged from .60 (for Psychological health) to .86 (for Environmental domain).

#### Procedure

Parents of children with ASD were contacted through diagnostic and therapeutic facilities specializing in ASD, or through psychological and pedagogical counselling centres, schools and parent associations. The institutions were asked to disseminate information about the study, and then those who gave consent to participate were contacted by telephone or in person and told in detail about the purpose and nature of the study. Parents were informed that the study was anonymous and that participants had the right to withdraw at any time. In this group, 95 pairs of questionnaires were distributed, with the order of scales in the sets randomized. Ten sets (10.5%) were sent by mail and 85 delivered personally. The questionnaires for mothers and fathers were placed in separate envelopes. In the instructions, parents were asked not to communicate with their partner while completing the questionnaires, and to place them in separate envelopes and seal them. Fifty-one pairs of questionnaires were returned either by mail or in person, giving a return rate of 53.7%. In most other cases parents informed us that time constraints prevented them from taking part in the study. Additionally, ten questionnaires were completed by mothers only, who informed us that due to work load or unwillingness to take part in research of this kind the father would not be participating in our project. Ultimately, 49 sets were included in statistical analyses (sets completed by two pairs were rejected due to their children exceeding the age limit). No sets were rejected in their entirety for incomplete data due to only very incidental gaps in information. When a substantial amount of information was missing from a scale (more than 10% of items), the results of that participant were ignored. This resulted in minor differences in group sizes across various statistical analyses.

In order to recruit parents of typically developing children we contacted primary, lower secondary and upper secondary. Seventy-one pairs of sets were distributed, five of which (7%) were sent by mail and the rest delivered in person. Similarly, mothers and fathers from the control group were asked to complete the questionnaires independently. The overall return rate was 79%, for a total of 56 pairs of sets, four of which were rejected (one due to cerebral palsy in one child and the remaining three due to the child being over 17 years of age). Again, no pairs of sets were rejected due to incomplete data, and missing information was handled in the same way as in the group of parents of children with ASD.

#### Ethics

All procedures performed in studies involving human participants were in accordance with the ethical standards of the institutional and/or national research committee and with the 1964 Helsinki declaration and its later amendments or comparable ethical standards. The project has been accepted by the Ethics Committee of the Faculty of Psychology at the University of Warsaw. According to local regulations, obtaining written consent from adult participants in studies not involving invasive or potentially stressful/harmful procedures is not required. Participants were informed about the voluntary nature of their participation.

#### Data analysis

On the basis of distribution analysis using the Kolmogorov-Smirnov test and kurtosis and skewness values, the decision was made to use parametric statistics. The groups of mothers and fathers of children with ASD and controls were compared using two-way analysis of variance (the first factor “group” had two levels–ASD vs Controls; the second factor was “parent’s gender”). A correction for multiple comparisons (Sidak/Bonferonni) was applied, and therefore in all comparisons and correlation analyses the p level was set at < .01. In order to estimate the strength of effects partial eta squared (η^2^) was calculated.

Pearson’s r correlation analysis was used to check correlations between assessments of family functioning and stress and QoL, and to investigate correlations between results in mother-father dyads. In order to estimate family functioning characteristics as a predictor of parenting stress and QoL, the Forward Entry Selection Regression Analysis method was used. Parenting stress (total score, child, family and personal domain) and quality of life (four domains) were taken as the dependent variables, while family functioning scores (General Scale, Dyadic Relationships Scale and Self-Rating scale total scores) served as predictors. Statistical calculations were made using SPSS v 21.

## Results

### Family functioning, parenting stress and quality of life–Groups comparisons (H1, H2, H3)

Descriptive statistics of the family functioning, parenting stress and QoL are shown in Table 2.

**Table 2:** Mean and standard deviations of mothers' and fathers' scores.

Variables	Parents of children with ASD			Parents of Controls		
	Total M (SD)	Mothers M (SD)	Fathers M (SD)	Total M (SD)	Mothers M (SD)	Fathers M (SD)
FAM–III						
General Scale	1.14 (.44)	1.19 (.47)	1.09 (.41)	.96 (.40)	.95 (.48)	.95 (.44)
Dyadic Relationships Scale	1.03 (.54)	1.09 (.57)	.97 (.51)	.85 (.42)	.85 (.45)	.85 (.43)
Self-Rating Scale	.92 (.35)	.98 (.34)	.95 (.34)	.75 (.29)	.83 (.36)	.79 (.33)
QRS–S						
Child	5.55 (2.94)	6.43 (2.90)	4.67 (2.73)	2.93 (2.21)	2.92 (2.02)	2.94 (2.39)
Personal	13.93 (3.83)	15.10 (4.06)	12.76 (3.21)	8.57 (2.83)	8.43 (2.80)	8.70 (2.89)
Family	7.69 (4.96)	8.41 (5.02)	6.98 (4.84)	3.96 (3.16)	3.84 (2.87)	4.08 (3.45)
Total score	27.17 (9.78)	29.94 (10.1)	24.41 (8.71)	15.56 (6.49)	15.34 (6.04)	15.78 (6.94)
WHOQoL-BREF						
Physical health domain	15.52 (2.46)	15.18 (2.62)	15.86 (2.27)	16.54 (2.12)	16.41 (2.40)	16.66 (1.82)
Psychological health domain	13.14 (2.68)	12.95 (2.93)	13.33 (2.42)	14.44 (2.31)	14.12 (2.23)	14.76 (2.38)
Social relationships domain	13.76 (3.32)	13.69 (3.31)	13.84 (3.37)	15.53 (2.92)	15.53 (3.19)	15.54 (2.67)
Environmental domain	14.09 (2.72)	13.90 (2.98)	14.29 (2.45)	14.57 (2.16)	14.33 (2.05)	14.82 (2.26)

M–Mean; SD–standard deviation; FAM III–family functioning, QRS-S–parenting stress, WHOQoL-BREF–quality of life

The results of ANOVA conducted in order to analyse the effects of parents’ group and gender on family functioning, parenting stress and QoL are presented in Table 3.

**Table 3:** Parents' group and gender effects on family functioning, parenting stress and quality of life.

Variables	Statistical Effect	Effect Description
FAM-III		
General Scale	Main effect of child diagnostic group F(1.189) = 8.370; p < .01; η^2^ = .043	Parents of children with ASD > parents of controls
Dyadic Relationships Scale	NS	
Self-rating Scale	Main effect of child diagnostic group F(1.199) = 10.287; p < .01; η^2^ = .049	Parents of children with ASD > parents of controls
QRS–S		
Child	Main effect of child diagnostic group F(1,198) = 53.298; p < .001; η^2^ = .061	Parents of children with ASD > parents of controls
Personal	Main effect of child diagnostic group F(1.196) = 132.121; p < .001; η^2^ = .041	Parents of children with ASD > parents of controls
	Interaction effect: parents’ gender x child diagnostic group F(1.196) = 7.870; p < .01; η^2^ = .039	Mothers of children with ASD > fathers of children with ASD; mothers of controls = fathers of controls
Family	Main effect of child diagnostic group F(1.198) = 40.583; p < .001; η^2^ = .17	Parents of children with ASD > parents of controls
Total score	Main effect of child diagnostic group F(1.193) = 99.306; p < .001; η^2^ = .34	Parents of children with ASD > parents of controls
WHOQoL-BREF		
Physical health domain	Main effect of child diagnostic group F(1.201) = 9.849; p < .01; η^2^ = .047	Parents of children with ASD < parents of controls
Psychological health domain	Main effect of child diagnostic group F(1.201) = 13.559; p < .001; η^2^ = .064	Parents of children with ASD < parents of controls
Social relationships domain	Main effect of child diagnostic group F(1.198) = 15.806; p < .001; η^2^ = .074	Parents of children with ASD < parents of controls
Environmental domain	NS	

NS–non significant

The main effects of “group” were demonstrated in two of the family functioning scales. Parents of children with ASD scored significantly higher (indicating lower family functioning) than parents of typically developing children in General and Self-Rating scales. There were neither main effects of “parent’s gender” nor interaction effects across all variables, and no group differences in Dyadic Relationships Scale.

“Group” and “parent’s gender” interaction effect was only confirmed for parenting stress in personal domain. Mothers of children with ASD scored higher than fathers, while no gender differences were found in the group of parents of typically developing children.

With respect to QoL, parents of children with ASD scored lower than parents of typically developing children in the following domains: physical, psychological and social relationships; no differences were found in the environmental domain.

### Family functioning, parenting stress and quality of life: Correlation analysis and regression analysis (H4 and H5)

In order to analyse the relationships between family functioning and adjustment measures–parenting stress and QoL, Pearson’s correlation coefficients were calculated. As gender differences were only present in parenting stress, further analyses were conducted on joint groups of parents of children with ASD and controls (Table 4).

**Table 4:** Correlations between family functioning and adjustment variables in the whole sample (N = 202)*.

Family functioning	Parenting stress				Quality of life			
	Child	Family	Personal	Total	Physical Health	Psychological	Social Relationships	Environmental
General	.270	.394	.331	.378	-.412	-.534	-.522	-.439
Dyadic Relationships	.232	.304	.214	.288	-.373	-.414	-.507	-.376
Self	.210	.400	.283	.355	-.357	-.452	-.517	-.374

* All coefficients listed in the table are statistically significant at the level of p < .01

Statistically significant correlations were present between three dimensions of family functioning and all adjustment measures (parenting stress and QoL). The more unfavourably parents assessed family functioning, the higher levels of parenting stress and the lower QoL they declared. All correlations were weak or moderate, the strongest being the correlation between the assessment of family functioning and quality of life with respect to social relationships and psychological domains.

In order to understand whether family functioning is a predictor for parenting stress and QoL, regression analyses on the entire sample with group differences as factor were conducted. The results are shown in Table 5.

**Table 5:** Group (ASD vs control) and family functioning as predictors for parenting stress and quality of life.

Predictors	QRS-S Child	QRS-S Family	QRS-S Personal	QRS-S Total	QoL Physical Health	QoL Psychological	QoL Social Relationships	QoL Environmental
Group	B = - .421	B = -.341	B = -.583	B = -.53		B = .13	B = .142	
	t = - 6.644	t = - 5.597	t = - 10.654	t = - 9.632		t = 2.182	t = 2.453	
	p < .001	p < .001	p < .001	p < .001		p < .05	p < .05	
FAM III								
General Scale		B = .18	B = .171	B = .168	B = -.241	B = -.36	B = -.238	B = -.292
		t = 2.259	t = 2.4	t = 2.341	t = -2.856	t = -4.622	t = -3.155	t = -3.469
		p < .05	p < .05	p < .05	p < .01	p < .001	p < .01	p < .01
Dyadic Relation-ships Scale					B = -.168		B = -.225	
					t = -2.03		t = -3.047	
					p < .05		p < .01	
Self-Rating Scale		B = .214					B = -.208	
		t = 2.606					t = -2.669	
		p < .05					p < .01	
Final statistics	R^2^ = .251	R^2^ = .306	R^2^ = .441	R^2^ = .434	R^2^ = .218	R^2^ = .333	R^2^ = .384	R^2^ = .224
	F (4,201) = 16.471	F (4,201) = 21.687	F (4,201) = 38.89	F (4,201) = 37.832	F (4,201) = 13.748	F (4,201) = 24.579	F (4,201) = 30.265	F (4.201) = 14.189
	p < .001	p < .001	p < .001	p < .001	p < .001	p < .001	p < .001	p < .001

B–Standardized Coefficients Beta

In the case of parenting stress, the “group” factor was a predictor for all measured aspects of stress. Overall family functioning was associated with the general stress level, as well as with family and personal parenting stress. In addition, the Self-Rating scale score was associated with family stress.

Overall family functioning was a predictor for the levels in all QoL domains. The Dyadic Relationships scale score was a predictor for physical health and social relationships domains, while the score on the Self-Rating scale–for the social relationships domain. The “group” factor was predictive of scores in the psychological and social relationships QoL domains.

### Consistency of mothers’ and fathers’ assessments of family functioning, level of stress and quality of life (Question 3)

Table 6 shows the results of correlation analysis in parent dyads conducted to find out whether mother and father offer similar assessments of family functioning, parenting stress and QoL.

**Table 6:** Correlations of partners’ assessments of family functioning, parenting stress and quality of life among parents of children with ASD and parents of controls.

Variables	Parents of children with ASD	Parents of controls
FAM–III		
General Scale	.707*	.388*
Dyadic Relationships Scale	.753*	.470*
Self-Rating Scale	.626*	.338*
QRS–S		
Child	.593*	.239
Personal	.263	.022
Family	.738*	.357
Total score	.599*	.132
WHOQoL-BREF		
Physical health domain	.328	.281
Psychological health domain	.151	.212
Social relationships domain	.467*	.401*
Environmental domain	.532*	.384*

* p < .01

In the parents of the children with ASD group statistically significant strong correlations were found between partners in their assessments of family functioning across all analysed scales (General, Dyadic Relationships, Self-Rating). In the control group there were also significant correlations, although they were weaker than in the parents of the children with ASD group.

Stress levels in mothers and fathers of children with ASD were moderately correlated in the child and family domains, while in the personal domain the correlation was not significant. In the control group there were no significant correlations in QRS domains. With respect to QoL, partners' results were significantly correlated in both study groups in two domains (social relationships domain and environmental domain).

## Discussion

The present study focused on the assessment of family functioning and its relationship with parenting stress and quality of life in parents of children with ASD without intellectual disability. We were also looking for gender differences in experiences of parenting in this group of parents, compared to parents of typically developing children, matched for family system characteristics. Generally, the most striking were the differences between parents of children with ASD and those of typically developing children in parental assessments of family functioning, parenting stress and QoL. Additionally, there was the interaction effect in personal stress that involved mothers of children with ASD reporting higher levels of stress than the fathers of these children, while no differences in stress levels between mothers and fathers were found in the control group. There were no gender differences in the assessment of family functioning and quality of life. The consistency of assessment of family functioning, parental stress (except personal stress) and QoL (social relationships and environmental domains) in parent dyads has been shown.

### Between-groups comparisons of family functioning

As predicted, parents of children with ASD reported lower levels of family functioning than controls. This was true for the General Scale and Self-Rating Scale, but not for the Dyadic Relationships Scale of FAM-III.

Lower assessments of family functioning by parents of children with ASD seems to be consistent with the results of those studies in which this group of parents reported lower family cohesion and adaptability compared to controls [61], and their family's expressive feelings as lower due to their child's communication difficulties [33]. It may be associated with the additional childcare burden on parents of children with ASD and perceived insufficiency of resources required to pay more attention to other family members and make efforts to satisfy their needs as well as the sense of neglecting other responsibilities, such as providing financially for the family, maintaining social relations and organising leisure and recreation [47,49].

We found no statistically significant difference between parents of children with ASD and controls in the satisfaction with dyadic relationship quality. This result seems not to be consistent with earlier reports on parents of children with autism [49,61–65]. The pressures of raising a child with ASD can make caring for the quality of the relationship difficult and be a source of conflict between partners [66]. This is particularly important because marital support in this group is central to effective coping with stress [61], and affects the sense of parenting competence [67]. When considering the results of the present study it should be noted that differences in parental assessment of dyadic relationships emerged at the alpha level of p < .05 (when Bonferroni correction was applied, they sank below statistical significance). As such, any interpretation of this finding must necessarily be tentative.

As far as perception of their own functioning in the family, parents of children with ASD assessed it lower than parents of typically developing children. Less favourable perception of themselves as family members can also result from burdens associated with childcare and a sense of not investing enough in family relations. However, an alternative interpretation should be mentioned here. Research on broader autism phenotype (BAP) has shown that some parents of children with ASD exhibit characteristics typical for autism [68,69]. These characteristics are milder in parents than in individuals diagnosed with ASD and often include only some aspects of functioning, such as cognitive deficits, preference to be alone, insistence on sameness, reluctance to change or obsessive-compulsive symptoms [70,71]. It is possible some of the parents of children with ASD in the study may presented BAP traits influencing both their functioning in the family and their assessments of family functioning. Since BAP traits were not measured in the study, no conclusions can be drawn on these potential relationships, but this issue should be taken into consideration in future studies.

There were no differences between mothers and fathers in the present study in terms of total scores in the three family functioning scales. McStay et al. [29] also found no gender differences in the parental reports of family functioning in parents of children with ASD, contrary to the differences in the level of the adjustment of mothers and fathers. Results of our study remain consistent with their findings, it must be noted however, that we have limited our analyses to the three main scales of FAM-III. More detailed exploration of the results, including subscales scores, could bring additional information in this field.

### Between-groups comparisons in parenting stress and quality of life

As predicted, the parents of children with ASD scored much higher in parenting stress than parents of typically developing children. This finding confirms previous reports [11,72] and is certainly related not only to the challenges faced by the parents of children with ASD, but also to the unsatisfactory level of support offered to individuals with ASD (including high functioning ones) and their families. This is a common problem for many countries [45,73–75]. It should be noted that higher stress in parents of children with ASD in the family subscale of QRS is consistent with their less favourable assessments of family relations in FAM-III. The results confirm that challenges resulting from the developmental problems of children with ASD may negatively impact family life also in the case of parents of children without intellectual disability.

We found interesting interaction effects of parent's gender and diagnostic group in the personal parenting stress. Mothers of children with ASD scored higher than fathers, while no gender differences were found among parents of typically developing children. These results can be viewed as an outcome of greater involvement in childcare, education and developmental support in mothers of children with ASD. The similar picture was previously found by Benson et al. [34]. No significant differences between mothers and fathers were found in the other aspects of parenting stress, which is consistent with the lack of such differences in FAM-III total scores.

As expected, parents of children with ASD assessed QoL in most analysed domains (physical health, psychological and social relationships) lower than parents of typically developing children. Lower QoL may result from the burdens and limitations experienced by parents in connection with childcare and insufficient social support, both professional [46] and informal [76,77]. In turn, lower assessments in the physical health domain of QoL may be associated with specific difficulties related to taking care of a child with ASD, such as circadian cycle disturbances and sleep deprivation that result in parental fatigue [78], or particularly burdensome behavioral problems [40,79]. These results confirm the need of parents to receive the efficient support in the ASD child rearing.

Similarly to other studies [25,43], we found no differences between the mothers and fathers of children with ASD in any of the QoL domains. Since gender differences in this group of parents were present in terms of personal stress, further research could investigate factors that determine the levels of stress and the quality of life in mothers and fathers of children with ASD without intellectual disability. Perhaps personal burdens associated with raising a child with ASD reflected by the levels of personal stress in mothers could be explained by the way their parental role and its associated duties are defined. In that case they could affect the levels of parental stress in the domain of self-evaluation as parent, while being less important for the subjective QoL in other domains. It would also be interesting to explore dispositional correlates of subjective stress and QoL in mothers and fathers. Some relevant information was collected previously by Yamada et al. [42], who reported on differences in that matter in mothers and fathers of children with ASD, and on the effects of the husband-wife relationship on QoL in women, though not in men.

### Relationships between family functioning and measures of adjustment

Correlation analysis yielded a number of significant relationships between family functioning and measures of parental adjustment. A positive correlation was found between assessment of family in all aspects (General Scale, Dyadic Relationships Scale, Self-rating Scale) and QoL, and a negative one between assessment of family and parenting stress.

In regression equations group (ASD vs control) and family functioning served as predictors for parental adjustment. In the case of parenting stress, group was a stronger predictor than any of the domains of family functioning. This finding is hardly surprising in the context of between-groups comparisons which confirmed that parents of children with ASD without intellectual disability experience much higher levels of stress than parents of typically developing children. Nevertheless, family functioning was also related to parental stress levels. FAM-III General Scale scores predicted both the overall stress level and the levels of stress in two domains (family and personal). The factors “group” and FAM III General Scale scores combined accounted for over 40% of variance in scores with respect to the overall stress level and personal stress. This finding confirms the importance of family relations for the level of stress experienced by parents of children with ASD without intellectual disability. Similar results were reported by McStay and colleagues [29]. They found that a significant predictor of parenting stress (explaining 8% in mothers and 5% in fathers) is family sense of coherence, and there was a correlation-level relationship between marital support and parenting stress in mothers. Jones and Passey [80] also demonstrated that integration and cooperation in the family explained the greatest portion of variance in parenting stress in parents of children with ASD (15%).

Family functioning measured in FAM III General Scale was also associated with all QoL domains. The result in Dyadic Relationships was predictive of quality of life in physical health and social relationships; in addition, Self-Rating was a predictor for social-relationships. These findings should be taken into account when designing support for the families of children with ASD, e.g. by providing families with opportunities to spend time or engage in leisure activities together.

It should be noted, however, that the relationships analysed in this paper are likely to be more complex than it appears from our results. Family functioning may be affected by a child’s developmental disorder, but it can also have an impact on how the child develops [81–83]. Kelly and colleagues [83] showed that in children with Asperger’s syndrome the quality of family relationships (in particular conflict in the family) is predicative of depressive and anxiety symptoms. Presumably, increased levels of depression and anxiety in the child may, in turn, affect family relations by increasing tension, consequently resulting in a greater number of conflicts. This type of circular relationship is difficult to identify in correlational and cross-sectional studies. The focus in this study was on the relationships between family functioning and parental adjustment, with the former treated as a predictor of parental stress and QoL. However, family functioning may be treated not as a predictor but as a measure of family adjustment. This is how some researchers have approached relationship quality [84], family quality of life [29] and marriage quality [31]. Thus, we can assume that the results of this study suggest a presence of relationhips between family functioning and parental stress and QoL in the group of parents of children with ASD without intellectual disability, but caution is highly recommended when drawing conclusions on the direction of the causal relationships between these variables.

### Correlations in partner dyad assessments

The last issue analysed in this study was consistency of assessment within parent dyads. It was shown that parents of children with ASD rated the functioning of their families similarly, with Pearson’s r for total scores ranging from .626 (Self-Rating Scale) to .753 (Dyadic Relationships Scale). This result is consistent with findings reported by Altiere and von Kluge [85], in which mothers and fathers of children with ASD assessed the cohesion and adjustment of their families similarly. The correlations in assessments of family by parents of typically developing children were also significant, although weaker (from .338 for Self-Rating Scale to .470 for Dyadic Relationships Scale).

The scores of mothers and fathers of children with ASD also correlated in parenting stress (excepting the personal domain). The absence of significant correlations in the personal domain suggests differences in the way mothers and fathers experience the personal costs of childcare. It is worth remembering here that with respect to this aspect of parental stress, in the group of parents of children with ASD mothers scored higher than fathers, while no gender differences were observed in controls. The underlying causal factor may be that mothers are more likely to give up their careers and individual development to become the primary carer of their child with ASD [86].

In terms of QoL, the assessments of mothers and fathers in the two groups were correlated in two domains: social relationships and environmental (moderate correlations). As in previous studies [40,41], partners’ assessments were not found to be correlated in QoL relating to the physical health and psychological domains. A similar pattern of QoL scores emerged in the group of parents of typically developing children. It seems therefore, that there is no need to analyse it as a function of the specificity of parents of children with ASD.

## Implications and limitations

The study design puts limitations on our ability to make cause-and-effect conclusions regarding the relationships between analysed variables. In contrast, longitudinal studies would enable us to trace the dynamics of changes in the family system and their consequences for the family members' adjustment. Furthermore, for the sake of clarity we have not analysed the relationships between parenting stress and QoL, even though they too are interesting and have been previously explored [43].

Some variables that were not accounted for in the study may significantly affect parental adjustment, such as severity of child’s behavioral problems, as well as the pile-up of demands not related to childcare. Moreover, the questionnaires were completed by partners raising children together. This approach has its advantages (more information about correlations inside the family), but is not free of limitations. Participation of parent dyads exclusively precludes generalization of results to single parents, who make up a sizeable group in the case of raising children with ASD [87]. Future studies could benefit from including the perspectives of other family members, in particular healthy siblings. In addition, the study only enrolled parents of children with ASD within intellectual norms. This means that the results may not be valid for parents of children with autism and intellectual disability [41]. The majority in the study were parents with secondary or higher education. This also limits the possibility of generalization. It should also be noted that only about 54% of parent dyads that originally declared willingness to fill in the questionnaires actually took part in the study. In most cases parents explained their withdrawal from the project by time constraints and this was especially true of fathers. Other researchers have mentioned the need for a better understanding of fathers' experiences associated with raising a child with ASD [88] and poor submission rates in fathers in the present study may support this view. It should be mentioned that submission levels around 50% have been reported in others studies on QoL in parents of children with ASD [43]. In the present study only sets completed by pairs of parents were included in the analysis; those completed by mothers only were excluded. The collection of full sets of instruments completed both parents presented a great challenge, increasing the percentage of attrition and complicating potential generalization of results.

Moreover, establishing contact with a significant proportion of parents through therapy and support centres for children with ASD probably skewed the composition of the group towards greater participation of individuals benefiting from professional support than in the general population of these families. Finally, the relatively small groups of parents in the study constitute another of its limitations.

Limitations notwithstanding, the study generated information that expands our knowledge of the functioning of families of children with ASD with normal-range intelligence and relationships between that functioning and measures of parental adjustment. This may lead to a better understanding of the conditions prevailing in the families in which these children develop. There is no doubt, however, that many questions in this area remain unanswered. They relate to factors affecting the functioning of families of individuals with ASD without intellectual disability, including social attitudes to people with autism, relationships between parents’ BAP traits and family functioning, as well as possible differences in the functioning of family systems with children with ASD with or without intellectual disability in the child.

## Supporting information

S1 Material Row data of all analyzed variables. (CSV)
